# Heavily treatment-experienced patients with HIV: are new mechanisms of action enough?

**DOI:** 10.1177/03000605241301883

**Published:** 2024-12-05

**Authors:** Marisa B. Brizzi, Tracy L. Cable, Dimple C. Patel, Kelli Williams, Zoe Adjei, Carl J. Fichtenbaum

**Affiliations:** 1 HIV Clinical Pharmacy Specialist, Department of Pharmacy, University of Cincinnati Health, Cincinnati, OH, USA; 2 Assistant Professor of Medicine, Division of Infectious Diseases, 12303 University of Cincinnati College of Medicine, Cincinnati, OH, USA; 3 Associate Professor of Medicine, Division of Infectious Diseases, 12303 University of Cincinnati College of Medicine, Cincinnati, OH, USA; 4 PGY-2 Ambulatory Pharmacy Resident, Department of Pharmacy, University of Cincinnati Health, Cincinnati, OH, USA; 5 Professor of Clinical Medicine, Division of Infectious Diseases, 12303 University of Cincinnati College of Medicine, Cincinnati, OH, USA

**Keywords:** HIV, antiretroviral therapy, heavily treatment-experienced people with HIV, HIV drug resistance, HIV salvage therapy, AIDS

## Abstract

Antiretroviral (ARV) drug resistance poses a threat to ending the HIV epidemic. As the rates of integrase resistance continue to increase globally, the availability of options for HIV treatment becomes limited. Heavily treatment-experienced (HTE) people with HIV (PWH) are limited to two or fewer available fully active ARV classes and are more likely to have an AIDS-defining event. Appropriate identification and management of HTE PWH is crucial to improving patient outcomes and reducing the future spread of drug-resistant HIV. As treatment options become more limited owing to drug resistance, the availability of more potent drugs with a marked increase in virologic suppression is needed in the current ART era. The purpose of this narrative review is to review the identification of HTE PWH, novel mechanisms of resistance, and management of HTE PWH in resource-rich and resource-limited settings using novel ARVs and combination ART.

## Introduction

Antiretrovirals (ARV) have changed the natural history of the global HIV epidemic. In 1995, potent combination antiretroviral therapy (ART) demonstrated the ability to suppress HIV replication, reconstitute the immune system, and allow people with HIV (PWH) to live longer.^1–2^ Currently, there are seven classes of ARVs approved for the treatment of HIV. However, exposure to ARVs over time can lead to resistance and drug-related toxicities. Additionally, drug–drug interactions can limit effective treatment options. Heavily treatment-experienced (HTE) PWH are so classified because they are limited to two or fewer of the available fully active ARV classes.^3^ Prior definitions of HTE based upon the number of previously used ART regimens are outdated because standard of care now includes switching regimens in PWH with a suppressed viral load to modernize therapy. Thus, the definition of HTE should be based upon prior treatment failure of ART, with or without documented drug resistance or documentation of drug resistance to more than two ARV classes.

The prevalence of HTE PWH varies based upon the definition used and among global regions. This prevalence has also changed over time, declining over the two decades since the advent of ART that is less toxic and easier to administer. The sharpest reduction in the prevalence of HTE PWH came after 2006 with the introduction of integrase inhibitors (INSTIs). In the United States (US), rates of HTE PWH declined from 7.5% to 1.8% between 2006 and 2007 with the approval of raltegravir (RAL).^4^ Despite this improvement in the US, rates of HTE PWH have not declined globally; for example, these rates have increased in Europe, from 6% in 2010 to 9% in 2016.^5^ Since 2018, the World Health Organization (WHO) has recommended dolutegravir (DTG)-based ART as the first-line regimen in PWH, with 91% of reporting countries adopting this guidance by 2023.^6^ Following the expansion of DTG use globally, reports of DTG resistance have been 4% to 6% worldwide, varying between low- and middle-income versus middle-high- and high-income countries.^7^ In PWH receiving DTG-based ART who have an unsuppressed viral load, rates of DTG resistance range from 3.9% to 19.6% in low- and middle-income countries versus 4.8% in high-income and upper-middle-income countries.^6^ Notably, DTG resistance can also emerge rapidly in the presence of an elevated viral load. In a study from South Africa among PWH with elevated viral loads and continued DTG exposure, the prevalence of DTG resistance increased from 2.7% in 2021 to 11.9% in 2022.^8^

Data on the rates of PWH with drug resistance mutations (DRMs) who are classified as HTE are limited to European and US cohorts. In the US, the HTE prevalence was estimated to range from 2% to 14% during 2016 to 2017.^9^ In Europe, rates of HTE prevalence increased from 6% to 9% between 2010 and 2016, with notable geographic variation observed between Eastern Europe (1%) and Western/Central Europe (16%). Within the US, as more potent treatment options have become available for HTE PWH, the prevalence of PWH with limited treatment options (≤2 available classes with ≤2 active drugs per class) declined from 5.2% to 7.5% during 2000 to 2006 to 1.8% in 2007, and remained <1% after 2012.^10^ Despite the benefits of new ARVs in the HTE population, there is a lag between introduction of new therapies to markets in high-income countries and their availability in low- and middle-income countries. For example, the INSTI DTG became available and recommended for use in 2013 in the US, but the WHO did not recommend DTG as part of the preferred first-line HIV treatment for all adults with HIV until 2018.^6^ In 2022, there were still an estimated 3.8 million PWH across five countries in sub-Saharan Africa where the HIV treatment guidelines did not recommend INSTI-based regimens.^11–12^

The clinical outcomes for HTE PWH are worse than those for people with less treatment experience, likely owing to incomplete suppression of HIV replication with the development of opportunistic complications and toxicities from ARVs required to treat extensively drug-resistant HIV. The OPERA cohort compared clinical outcomes between HTE PWH and non-HTE PWH and demonstrated that HTE PWH were less likely to achieve virologic suppression, less likely to maintain CD4 counts over 200 cells/mm^3^, more likely to change regimens, and more likely to have an AIDS-defining event.^9^ Regimens to treat HTE PWH are more complicated and may require the use of ARVs with a lower barrier to resistance compared with first-line therapies. This is problematic because the lack of sufficient adherence and drug intolerance are key contributors to the development of virologic failure.^13^ Complicated regimens with more pills administered multiple times a day and with a lower barrier to resistance creates conditions that can lead to more treatment failures. Thus, the challenge of managing HTE PWH often requires extra resources, support, and monitoring to ensure success. The purpose of this narrative review was to review the identification of HTE PWH, novel mechanisms of resistance, and management of HTE PWH in resource-rich and resource-limited settings using novel ARVs and combination ART.

## Methods

All study types (primary reports and systematic reviews) were included within the literature search, with priority given to randomized controlled trials or meta-analyses. We used the search terms “heavily treatment-experienced,” “multi-drug resistant,” “salvage therapy,” “treatment failure,” “cART-experienced,” and “antiretroviral experienced” to search articles in PubMed from January 2009 to January 2024. More than 1800 papers were extracted. Among the articles published during the search period, we selected 40 papers of interest related to the treatment of HTE PWH with HIV. We also reviewed international guidelines on the management of HIV and ClinicalTrials.gov for ongoing studies evaluating investigational agents. The nature of this study precludes the requirement for both ethics approval and informed consent because this was a review based on the published literature.

## Identification of HTE PWH

### Mechanisms of drug resistance

HTE PWH are classified as those diagnosed with an HIV virus that has DRMs to multiple antiretroviral medications, typically having two or fewer antiretroviral classes available for use with limited fully active agents within each class.^3^ HIV DRMs are caused by mutations in the viral genome that affect the ability of ARV drugs to inhibit HIV replication.^6^ DRMs are acquired via two pathways: transmitted drug resistance (TDR) and acquired drug resistance (ADR). TDR is a type of HIV drug resistance that typically occurs when primary HIV infection is caused by a DRM-bearing virus. ADR is a type of HIV drug resistance that occurs when ongoing viral replication occurs in the presence of suboptimal drug levels. Individuals with pre-treatment drug resistance refers to those starting ART with either acquired (i.e., from ARV prophylaxis) and/or transmitted resistance prior to initiating first-line therapy. Most drug-resistant viruses are less fit than drug-susceptible viruses and will be replaced by wild-type virus over time in the absence of selective ARV pressure. Nearly all clinically important DRMs arise in the setting of selective drug pressure.^14^ The number of DRMs required to reduce susceptibility varies among ARVs and is directly tied to an ARV’s barrier to resistance. Some mutations, like M184V, are sufficient alone to reduce susceptibility to an ARV. Additionally, cross-resistance within ARV drug classes is common because most DRMs reduce susceptibility to multiple ARVs of the same class but will not impact susceptibility to an ARV in another drug class.

Historic sequential monotherapy treatment of HIV with ARVs that have a low barrier to resistance and low potency leads to the rapid development of DRMs. The most common mechanisms for ADR in PWH are non-adherence to ART and drug intolerance/toxicity. However, with modern ARVs used for treatment and prevention, new mechanisms of resistance are beginning to emerge.^13^ Patients receiving HIV pre-exposure prophylaxis (PrEP) may acquire pre-treatment drug resistance if they seroconvert while receiving PrEP, especially those on long-acting (LA) therapies. It is estimated that 20% of patients who seroconvert on emtricitabine/tenofovir disoproxil fumarate (FTC/TDF) will develop nucleoside reverse-transcriptase inhibitor (NRTI) DRMs.^6^ In a modeling study from sub-Saharan Africa, investigators estimated that 20 years after the introduction of cabotegravir (CAB-LA) PrEP, approximately 13% of ART initiators would have INSTI resistance versus 1.7% in the absence of CAB-LA PrEP; however, this would lead to a 29% decrease in the incidence of HIV and a reduction of 4540 AIDS deaths per year over 50 years.^15^

A newer emerging mechanism of resistance is the presence of insufficient concentrations of ARVs despite adequate adherence. Resistance owing to insufficient concentrations has been documented in patients receiving LA injectables for HIV treatment. In a cohort of PWH who had previously been suppressed on oral ART without known risk factors for virologic failure and who switched to CAB/rilpivirine (CAB/RPV-LA), there were five virologic failures with DRMs among patients with pharmacokinetic data demonstrating insufficient drug levels.^16^ The reasons for insufficient drug levels varied from a higher body mass index, inappropriate administration, and/or delayed injections. All five patients had acquired at least two reverse transcriptase (RT) resistance mutations and four had acquired at least one IN mutation at the time of failure. Lenacapavir (LEN), an LA therapy administered every 6 months, was associated with the acquisition of DRMs in eight participants (six with M66I, one with K70H, one with Q67H + K70R) who received unintended functional LEN monotherapy owing to non-adherence to additional ARVs at the time of resistance selection.^17^

### HIV-1 drug resistance testing

HTE PWH are identified via baseline genotyping (i.e., pre-treatment drug resistance) or in the presence of virologic failure (i.e., unidentified TDR or ADR).^13–14^ It is important to note that TDR accounts for 10% to 14% of drug resistance; therefore, genotyping should be performed at baseline prior to starting ART in newly diagnosed PWH. Baseline genotyping should include testing for genes encoding RT and protease (PR), and testing for genes encoding IN should be added in patients with suspected transmitted IN resistance or in patients who have a history of receiving CAB-LA for PrEP. Patients with persistent HIV-RNA levels ≥200 copies/mL while reporting adherence to ART are usually experiencing virologic failure, which is often associated with viral evolution and accumulation of DRMs.^13^ This association is particularly common when the HIV RNA level is >500 copies/mL. Evidence suggests that selection of DRMs does not occur in PWH with RNA levels that are persistently suppressed below the lower limit of detection (LLOD) of current assays.

Genotyping, phenotyping, and tropism assessments are the primary testing modalities used to identify resistance profiles.^13^ In the presence of virologic failure, HIV-1 drug-resistance testing should be performed while the individual is on the failing regimen or within 4 weeks of discontinuing an oral regimen. Given the extended half-lives of LA injectable ARVs, drug-resistance testing may still be useful beyond 4 weeks after discontinuation of LA therapy. Resistance testing that includes IN genes should be performed in patients who have experienced virologic failure on a regimen containing CAB/RPV-LA or those who have acquired HIV while receiving CAB-LA for PrEP, regardless of the amount of time since drug discontinuation. Drug-resistance genotype testing is recommended in PWH who have HIV RNA >200 copies/mL. It is important to note that PWH with RNA <500 copies/mL and certain HIV subtypes may not be able to have resistance detected owing to an inability to amplify the virus. In standard HIV genotype assays, this may occur in approximately 10% of individuals tested. A next-generation sequencing genotypic resistance assay that analyzes HIV-1 proviral DNA in host cells is now commercially available and can detect archived resistance mutations in PWH with RNA levels below the LLOD or with low-level viremia. Proviral DNA testing can be considered if genotypic resistance test results cannot be obtained owing to low RNA levels. Results from this test should be interpreted with caution because these assays might miss some or all previously existing DRMs. Furthermore, the presence of DRMs in proviral DNA does not always correlate with clinical treatment failure.^18^

Standard genotyping detects drug-resistance mutations for the three major HIV enzymes: RT, PR, and IN.^13^ Most assays involve conventional Sanger sequencing of these genes from circulating RNA in plasma to detect mutations that confer drug resistance. These results typically indicate which medications will be active or inactive; however, these assays do not typically consider combinations of mutations that may be synergistic or negating or those that may be required to interpret complex results. Genotypic testing is preferred over phenotypic testing to guide therapy in people with virologic failure while on first- or second-line regimens, particularly for people in whom mutation patterns are not expected to be complex. Phenotypic assays measure the ability of a virus to grow under different concentrations of ARV drugs. This is useful when many mutations are present to predict the anticipated degree of sensitivity to individual agents. Phenotypic assays require a longer turnaround time and are more expensive than genotyping; therefore, these tests should be reserved for PWH who are failing more than their second regimen. Note that phenotypic assays are most informative when a person is currently taking the failing regimen. The addition of phenotypic to genotypic resistance testing is recommended for people with known or suspected complex multi-class drug-resistance mutation patterns.

For ARVs with novel mechanisms of action, separate testing will need to be performed to determine susceptibility. Tropism testing determines which coreceptor the virus uses during entry into cells: CCR5 only, CXCR4 only, mixed, or both.^13^ Because the ARV maraviroc is a CCR5-coreceptor antagonist, it can only be used with viruses that use CCR5 alone. It is recommended that a tropism test be performed prior to maraviroc use. Unfortunately, there are limited commercially available testing options for novel therapies like ibalizumab (IBA), LEN, and fostemsavir (FTR).

## Management of HTE PWH

### Guideline recommendations

**Figure 1.**
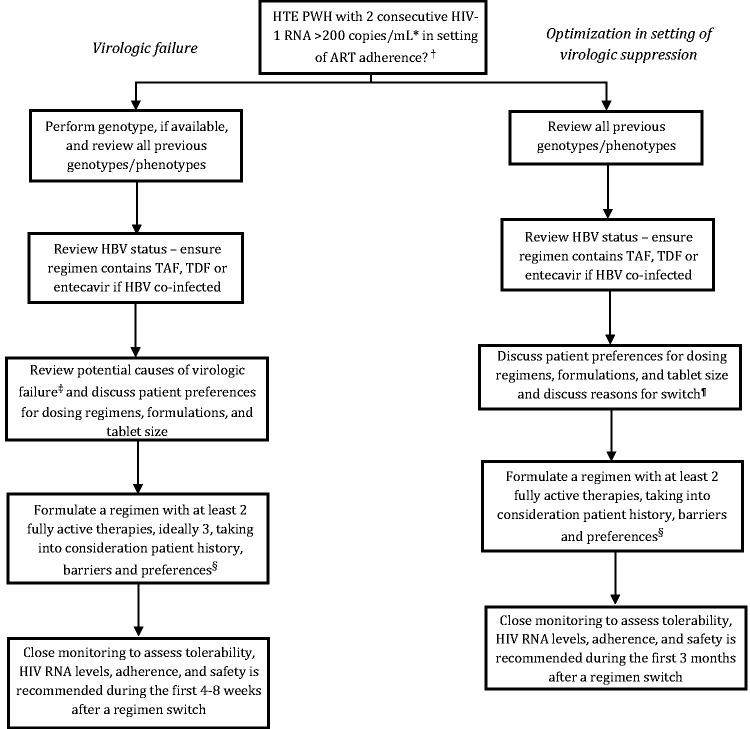
Treatment algorithm for management of heavily treatment-experienced people with HIV^13,19^ *>1000 copies/mL in resource-limited settings. ^†^Assessment of antiretroviral therapy (ART) adherence, drug–drug interactions, drug–food interactions, drug tolerability, HIV RNA level and CD4 cell count trends over time, ART history, co-existing medical conditions, previous ART, and all drug-resistance test results should be considered. ^‡^Non-adherence, pill burden, drug–drug interactions, drug–food interactions, drug adverse effects, affordability, and/or stigma-related concerns. ^§^In resource-limited settings, consider a regimen containing dolutegravir, boosted darunavir, and an optimized nucleoside reverse-transcriptase inhibitor backbone. ^¶^Simplification, modernization, drug–drug interactions, drug–food interactions, drug adverse effects, affordability, pregnancy, and/or stigma-related concerns.

The goal of treatment for HTE PWH who are experiencing virologic failure is to establish virologic suppression such that HIV replication ceases and does not allow for the development of additional DRMs.^13^ For some rare HTE PWH with extensive DRMs, virologic suppression may not be possible; thus, the new ART regimen should be designed to maintain CD4 counts, preserve treatment options, delay clinical progression, and minimize toxicity. When changing regimens in HTE PWH, there are two typical scenarios: changing ART in the setting of virologic failure and optimizing ART in the setting of virologic suppression. Figure 1 provides guidance on how to approach ART modification in either scenario.^13,19^

### Management of virologic failure

Virologic failure is defined as two consecutive results of HIV-1 RNAs >200 copies/mL in the setting of adequate ART adherence.^13^ The management of virologic failure must be individualized and expert advice can be critical. Additionally, the choice of ARVs will be driven by drug-resistance genotypes and/or phenotypes, ARV treatment history, drug tolerability, hepatitis B virus (HBV) coinfection, and patient preference. In HTE PWH with a limited available treatment history, every effort should be made to obtain historical records including prior genotypes or phenotypes. HTE PWH should receive regimens containing ARV agents with a high barrier to resistance, which are those in which emergent resistance is uncommon and include boosted darunavir (DRV), DTG, and bictegravir (BIC). A new regimen should include two fully active ARV drugs if at least one has a high barrier to resistance. If there is no fully active drug with a high barrier to resistance available, then every effort should be made to include three fully active drugs. Typically, when patients fail non-NRTI (NNRTI)-, INSTI-, or protease inhibitor (PI)-based first-line regimens containing an NRTI backbone, it is recommended to switch to a second-line therapy containing two NRTIs plus DTG, BIC, or boosted DRV, or to switch to a boosted PI and INSTI combination. Salvage regimens involving the use of three or more potent agents are typically used in ART-experienced individuals with multiple DRMs and few fully active treatment options.^19^ Although trials in HTE PWH often involve adding a single ARV to a failing regimen for a short period, this is not recommended as it may lead to the development of additional DRMs.^13^ Options for additional ARVs may be limited, but the entire ARV regimen should be reviewed and optimized when changing therapy in the setting of treatment failure. Discontinuing or briefly interrupting therapy in a patient with overt or low-level viremia is not recommended because it may lead to a rapid increase in HIV RNA and a decrease in CD4 cell count, which increases the risk of clinical progression. Studies have demonstrated that even partial virologic suppression of HIV RNA to >0.5 log10 copies/mL from baseline is correlated with clinical and immunologic benefits. Low-level viremia, defined as HIV RNA above the lower limit of detection and <200 copies/mL, typically does not require a change in treatment for HTE PWH; current ARV regimens should be continued with HIV RNA levels monitored at least every 3 months to assess the need for changes to ART in the future. Continuing ART reduces the risk of disease progression even in the presence of viremia and no improvement in CD4 count.

Multiple studies have reported the benefits of using ARVs with a high barrier to resistance, novel mechanism of action, or unique combinations in HTE patients experiencing virologic failure (Table 1).^20–27^ Early trials evaluating the use of novel ARVs in the setting of treatment failure in HTE PWH involved adding a new ARV to a failing regimen.^20–22^ In 2007, the DUET 1 and 2 clinical trials demonstrated the efficacy of etravirine (ETR) and DRV/ritonavir (RTV) in combination for multidrug-resistant strains of HIV.^20–21^ In the 2008 BENCHMRK trial, the first INSTI—RAL—demonstrated efficacy in PWH with limited treatment options.^22^ In 2009, the ANRS 139 TRIO Trial demonstrated safety and efficacy of an NRTI-sparing regimen containing three new drugs: ETR, RAL, and DRV/RTV, in a population of HTE PWH experiencing treatment failure.^23^ The ANRS 139 TRIO Trial set the stage for future studies to evaluate switching of ARV regimens to novel and unique combinations in the setting of treatment failure among HTE PWH. Historically, it has been recommended to continue NRTIs in salvage regimens, even in the presence of NRTI resistance, owing to some mutations (i.e., M184V) reducing viral fitness and increasing susceptibility to other NRTIs and delaying emergence of additional NRTI mutations (i.e., thymidine analogue mutations).^13,19^ The OPTIONS trial further highlighted that salvage regimens do not need to contain NRTIs, and optimized regimens should be selected on the basis of treatment history and susceptibility testing.^25^ HBV/HIV coinfection will affect ARV choices, with the salvage regimen needing to contain tenofovir alafenamide (TAF) or TDF to avoid HBV rebound and hepatocellular damage.^13,19^ FTC and lamivudine (3TC) are not recommended as first-line HBV treatments and should not be used alone in HBV coinfection. Entecavir may be used if TAF or TDF are not options owing to HBV resistance or potential adverse effects. Close monitoring to assess tolerability, a reduction in HIV RNA, adherence, and safety is recommended during the first 4 to 8 weeks after a regimen switch in the setting of virologic failure.^13^

**Table 1.** Review of major historical trials using novel combinations in heavily treatment-experienced PWH^20–25,27–30^

Study	Treatment	Design	Population	Outcomes
*In the setting of treatment failure*				
ANRS 139 TRIO Trial*France*2009^23^	DRV/r + ETR + RAL	Phase II, noncomparativemulticenter trial	HIV-1 RNA ≥1000 copies/mL while receiving ART for ≥8 weeks; ≥3 PI- and NRTI- RAMs but susceptible to DRV	48 weeks:86% (89/103) (95% CI, 80–93) had HIV-1 RNA <50 copies/mL. The median CD4 cell count increase was 108 cells/mm^3^
ETR + MVC + RAL*Italy*2010^24^	ETR + MVC + RAL	Single-center, prospective, cohort	PWH failing current ART with exposure to NRTIs, NNRTIs, and PIs and harbored R5 virus.	48 weeks:100% (28/28) had HIV-1 RNA <400 copies/mL; 93% (26/28) had HIV-1 RNA <50 copies/mL
OPTIONS*USA*2015^25^	Optimized regimen + NRTIs (add-NRTIs group) vs. optimized regimen without NRTIs (omit-NRTIs group)	Multicenter, randomized, controlled trial	HIV-1 RNA ≥1000 copies/mL, history of PI-based regimen, history of NRTI- and NNRTI-RAMs	48 weeks:360 participants included and 93% completed a 48-week visit. Cumulative probability of regimen failure was 29.8% in the omit-NRTIs group vs. 25.9% in the add-NRTIs group (95% CI, −6.1 to 12.5%)
TIVISTA*Italy*2017^26^	DRV/b + DTG	Multicenter, observational cohort	PWH switched to DRV/b + DTG; 88.6% with NRTI-RAMs, 72.6% with NNRTI-RAMs, 68.1% with PI-RAMs, 10.6% with INSTI-RAMs	24 weeks:Proportion of PWH with HIV-1 RNA >50 copies/mL declined from 43.4% (49/113) to 6.2% (7/113)
NADIA*Kenya, Uganda, Zimbabwe*2022^27^	DTG + FTC/TDF, DTG + FTC/ZDV, DRV/r + FTC/TDF, DRV/r + FTC/ZDV (randomized 1:1:1:1)	Prospective, multicenter, open-label, randomized, non-inferiority trial	HIV-1 RNA ≥1000 copies/mL while receiving an NNRTI-based regimen for ≥6 months	96 weeks:90% (211/235) in DTG group vs. 87% (199/229) in DRV group had HIV-1 RNA <400 copies/mL (p=0.33)
*In the setting of suppressed optimization*				
BITER Study*Spain*2014^30^	DRV/r + ETR	Multicenter retrospective, observational cohort	HIV-1 RNA <1000 copies/mL switched to DRV/r + ETR	24 weeks:89% (81/90) had HIV-1 RNA <50 copies/mL
TivEdO*Italy*2016^31^	DTG/RPV	Multicenter retrospective, observational cohort	PWH switched to DTG/RPV; 45.5% with RT-RAMs, 44.7% with PI-RAMs, 0.8% with INSTI-RAMs; 88% with HIV-1 RNA <50 copies/mL	Median 24 months:99% (131/132) had HIV-1 RNA <50 copies/mL
EVG/c/FTC/TAF + DRV*USA and Canada*2017^32^	EVG/c/FTC/TAF + DRV vs. baseline regimen (randomized 2:1)	Multicenter, phase 3, open-label, randomized trial	HIV-1 RNA <50 copies/mL on DRV/RTV-containing regimen for ≥4 months; with documented resistance to ≥2 ARV classes	24 weeks:96.6% (86/89) vs. 91.3% (42/46) (difference 5.3%, CI: −3.4% to 17.4%) had HIV-1 RNA <50 copies/mL
BIC/FTC/TAF + DRV/c*Spain*2023^33^	BIC/F/TAF + DRV/c	Multicenter, single-arm, open-label, Phase IV, pilot trial	HIV-1 RNA <50 copies/mL on 3-ARVs from three different classes owing to ARV failure	48 weeks:95% (60/63) had HIV-1 RNA <50 copies/mL
The DoDo Experience*Germany and Austria*2023^28^	DTG + DOR	Multicenter, single-arm, observational trial	HIV-1 RNA <50 copies/mL in all but three patients; 38% had PI-RAMs, 51% had NRTI-RAMs, and 35% had NNRTI-RAMs	Median of 265 days:88% (75/85) remained on DTG + DOR with HIV-1 RNA <50 copies/mL. 12% (10/85) switched off DTG + RPV: five owing to adverse effects, two switched to another 2DR, one owing to provider concern about previous DRV-RAM, and two owing to LLV

PWH, people with HIV; DTG, dolutegravir; DOR, doravirine; PI, protease inhibitor; NRTI, nucleoside reverse-transcriptase inhibitor; NNRTI, non-NRTI; ARV, antiretroviral; BIC, bictegravir; TAF, tenofovir alafenamide; DRV, darunavir; c, cobicistat-boosted; RTV, ritonavir; FTC, emtricitabine; CI, confidence interval; RPV, rilpivirine; INSTI, integrase inhibitors; ETR, etravirine; TDF, tenofovir disoproxil fumarate; r, ritonavir-boosted; RAL, raltegravir; ZDV, zidovudine; b, booster; MVC, maraviroc; LLV, low-level viremia; RAM, resistance-associate mutation; EVG, elvitegravir.

### Optimizing regimens in virologically suppressed patients

HTE PWH may choose to optimize their ARV regimen in the setting of virologic suppression to reduce toxicity, drug–drug or drug–food interactions, pregnancy, pill burden, or to simplify dosing.^13^ The goal of ARV optimization is to maintain a suppressed viral load while avoiding compromising future ARV options. When optimizing an ARV regimen in the setting of virologic suppression, previous genotypes and/or phenotypes, ARV treatment history, drug tolerability, HBV coinfection, and patient preference should still be reviewed prior to the switch. Some HTE PWH may desire simplification of ART if they are receiving multi-tablet regimens. In the DoDo Experience observational study evaluating DTG + doravirine (DOR) in treatment-experienced PWH, reasons for switching to a simplified two-drug regimen (2DR) included tolerability of the preceding regimen, drug–drug interactions, and reducing long-term adverse effects.^28^ Additionally, some HTE PWH may desire to switch to an LA injectable regimen for various reasons; this should be considered in HTE PWH who are virologically suppressed with no RPV or CAB DRMs, no HBV coinfection, and who are engaged in their care and able to show up for frequent clinic visits for administration. In the SOLAR study comparing every-2-month CAB/RPV-LA to oral BIC/FTC/TAF, 90% of patients in the CAB/RPV-LA arm preferred LA therapy.^29^ Reasons for preference were not having to worry about remembering to take HIV medication, convenience, not having to carry HIV medication, not having to think about HIV status daily, and not having to worry about others seeing or finding HIV medications.

Multiple studies have reported the benefits of using unique combinations of ARVs to optimize therapy in virologically suppressed HTE patients (Table 1).^28,30–33^ Even in the setting of virologic suppression, consultation with an HIV specialist is recommended when planning a regimen switch in an HTE PWH.^13^ Multiple studies have demonstrated the efficacy of novel combinations including single tablet regimens (STRs) in combination with other ARVs or unique NRTI-sparing dual therapies in virologically suppressed HTE patients.^28,30–33^ At our institution, in a small cohort of 32 virologically suppressed HTE PWH, we simplified the ARV regimen from a median six tablets/capsules to two tablets/capsules, while maintaining virologic suppression.^34^ When optimizing regimens in virologically suppressed HTE PWH, close monitoring to assess tolerability, viral suppression, adherence, and safety is recommended during the first 3 months after a regimen switch.^11^

### Practical management of HTE patients

When managing HTE PWH, it is important to discuss the history of ARV treatments and reasons for past failure together with the patient. Certain factors including toxicities, pill size, pill burden, dosing schedule, and route of administration should be discussed and considered when formulating a new regimen as these can affect treatment adherence. If wild-type virus is present on genotypic testing, this often indicates non-adherence to an ARV regimen.^14^ Patients who develop VF without DRMs can often be successfully treated with adherence counseling alone and resumption of ART. If new DRMs are identified, they should be reviewed, compiled with previous DRMs, and input to a rule-based algorithms, such as the Stanford University HIV Database, to provide a comprehensive interpretation of the results.^19^ When creating a salvage regimen, drug interaction checkers, like the HIV drug interaction checker from the University of Liverpool, should be used to ensure there are no drug–drug interactions among nontraditional combinations of ARVs, in addition to drug–drug interactions with concomitant therapies.^35^ In the setting of HTE PWH, fully active agents may be limited and ARV combinations may be required to formulate one fully active agent. Additionally, dosing may differ for agents used in the setting of resistance; both DRV boosted with ritonavir (DRV/r) and DTG are advised to be given twice daily when certain PI and INSTI mutations are present, respectively. Novel ARVs may also need to be used, like entry inhibitors or capsid inhibitors, where mechanisms of action differ between agents and resistance to one subclass does not confer resistance to others.^36^ Combinations of entry inhibitors can also be considered in the same regimen, and the route of administration should be considered based on patient preferences. Lastly, in HTE patients who transfer care between providers, every effort should be made to obtain previous information regarding genotypes and phenotypes. In the US, clinicians should be aware that some companies store old genotype records, and these may be available directly from the company website. If no historical genotypes or phenotypes can be obtained, a proviral resistance assay can be considered; however, the results should be interpreted with caution, as mentioned previously.

### Management of HTE patients in resource-limited countries

In resource-limited settings, dried blood spot testing is often used instead of plasma specimens owing to the limited availability of cold chain resources in health care facilities.^37^ Dried blood spot samples can be used for viral load testing at a treatment failure threshold similar to plasma samples at 1000 copies/mL. Therefore, in resource-limited settings, virologic failure is defined as two consecutive HIV-1 RNA levels >1000 copies/mL despite adherence to an ART regimen.^19^ Genotypes and phenotypes are often not available and not routinely performed at baseline or at time of virologic failure. The WHO recommends initiating therapy with DTG-based therapy and switching to ARVs with high barriers to resistance in the setting of virologic failure.^38^ If a patient fails an NNRTI-based regimen, it is recommended to switch to a DTG-based regimen, and if a patient fails a DTG-based regimen it is recommended to switch to a DRV-based regimen. If a patient fails a second-line regimen, the WHO recommends genotyping, if available, and recommends a salvage regimen containing DTG, boosted DRV, and an optimized NRTI backbone. For individuals with no active drugs available or in settings without access to ARVs with unique mechanisms of action for HTE patients, the WHO recommends continuation rather than cessation of ART.

## Contemporary therapies for HTE PWH

Contemporary ARV therapies with unique mechanisms of action and novel formulations provide additional options for HTE PWH with multiple DRMs.

### Ibalizumab (IBA)

IBA is a CD4-directed post-attachment HIV-1 inhibitor used in combination with an optimized background regimen (OBR) for the treatment of HIV in HTE adults with multidrug-resistant HIV-1 infection failing their current ARV regimen.^39^ Dosing and administration of IBA are summarized in Table 2. IBA non-competitively binds to the CD4 receptor after HIV attachment, preventing viral fusion and entry into cells. In phase 3 clinical trials, participants with HIV-1 RNA >1000 copies/mL and documented resistance to at least one ARV from each of three classes of ARV medications (NRTI, NNRTI, and PI) who received loading and maintenance doses of IBA showed a 1 to 2 log_10_ reduction in HIV-1 RNA over 25 weeks.^40^ At week 25, HIV-1 RNA <50 and <200 copies/mL was achieved in 43% and 50% of participants, respectively. An increase in the mean and median CD4+ T-cell count was also observed from baseline to week 25, increasing by 44 cells/mm^3^ and 17 cells/mm^3^, respectively. The most common adverse reactions reported in at least 5% of participants were diarrhea, dizziness, nausea, and rash.

**Table 2.** Dosing and administration of newly approved therapies for the PWH^39,41,43^

Antiretroviral	Mechanism of action	Dosing	Adverse reactions(incidence ≥5%)	Drug interactions
Ibalizumab IBA*(TROGARZO)*^33^	CD4 post-attachment inhibitor	Loading dose: IBA 2,000 mg IV × 1 doseMaintenance dose: IBA 800 mg IV every 2 weeksDosing window: ±3 days	Diarrhea, dizziness, nausea, and rash	None
FostemsavirFTR*(RUKOBIA)*^35^	gp-120 attachment inhibitor	FTR 600 mg PO twice daily	Nausea	Strong CYP3A inducers result in decreased concentrations of temsavir
Lenacapavir LEN*(SUNLENCA)*^37^	Capsid inhibitor	Dosing regimen 1:Day 1: Inject 927 mg SC and take 600 mg PODay 2: Take 600 mg PODosing regimen 2:Day 1: Take 600 mg PODay 2: Take 600 mg PODay 8: Take 300 mg PODay 15: Inject 927 mg SCMaintenance Dosing:Inject 927 mg SC every 6 months	Nausea and injection site reactions	Strong CYP3A4 inducers result in decreased lenacapavir plasma concentrations

PWH, people with HIV; IBA, ibalizumab; IV, intravenous; PO, per os; FTR, fostemsavir; SC, subcutaneous.

### Fostemsavir (FTR)

FTR is a pro-drug, with the active metabolite being temsavir, that binds to the envelope protein gp120 and prevents the conformational change required for attachment to the host cell’s CD4 receptors, thereby halting the viral entry process.^41^ Dosing and administration of FTR is summarized in Table 2. The phase 3 BRIGHTE trial reported the long-term safety and efficacy of FTR in HTE adults in combination with an OBR after 240 weeks.^42^ FTR maintained HIV-1 RNA <40 copies/mL in 45% of the randomized cohort, including participants with only one or two fully active ARV options, and 22% of the non-randomized cohort, including participants with no remaining fully active approved ARV options and combined with investigational agents. At week 240, the mean change from baseline in the CD4+ T-cell count was 296 cells/mm^3^ in the randomized cohort and 240 cells/mm^3^ in the non-randomized cohort. Decreased susceptibility to temsavir was reported in 70% of nonrandomized group participants in the BRIGHTE trial and was largely attributed to emergent substitutions in gp120 at four key sites.

### Lenacapavir (LEN)

LEN is a first-in-class capsid inhibitor that inhibits viral replication at multiple stages of the HIV life cycle including capsid-mediated nuclear uptake of HIV-1 proviral DNA, virus assembly and release, and capsid core formation.^43^ Dosing and administration of LEN are summarized in Table 2. The CAPELLA trial evaluated the safety and efficacy of LEN in HTE patients in combination with an OBR over 26 weeks.^44^ The study was divided into two cohorts: cohort 1 was the randomized cohort and included participants who had stable viremia and a lack of response to the failing therapy during the screening period; cohort 2 was the non-randomized cohort and included participants who had at least a <0.5 log_10_ copies/mL reduction in HIV-1 RNA level during the screening period. In cohort 1, patients were randomized 2:1 to receive LEN or placebo in combination with their failing regimen. The primary endpoint was a reduction in HIV-1 RNA of ≥0.5 log_10_ copies/mL by day 15, which was achieved by 88% of participants in the LEN arm compared with 17% in the placebo arm. At day 15, the failing regimen was changed to an OBR; at 26 weeks, 81% of patients in cohort 1 and 86% of patients in cohort 2 achieved HIV-1 RNA <50 copies/mL. Additionally, LEN demonstrated a least-squares mean increase in the CD4+ T-cell count of 75 cells/mm^3^ and 104 cells/mm^1^ in cohorts 1 and 2, respectively. Within both cohorts, eight patients in total developed resistance to LEN, with six patients developing a M66I mutation, all of whom had either inadequate OBR drug levels or no effective ARVs in the OBR.

### Pipeline

As the emergence of drug resistance continues to increase and contribute to ART failure, new mechanisms of action may not be enough. The development of new ARVs in HTE patients should focus on improved safety profiles, unique mechanisms of action, alternative routes of administration, extended dosing intervals, and fixed-dose combination therapies to improve adherence. ARTISTRY-1, an ongoing phase II/III study, is evaluating the efficacy of a unique fixed-dose combination of BIC+LEN in PWH receiving a complex ARV regimen owing to previous viral resistance, or intolerance, or contraindication to existing STRs.^45^ LATITUDE, a phase III study evaluating CAB/RPV-LA compared with daily oral therapy in 294 individuals with adherence challenges, demonstrated superior efficacy at maintaining virologic suppression in the LA injectable arm.^46^ Novel ARVs with unique mechanisms of action and extended dosing intervals are also being developed for HTE PWH. The safety and efficacy of a weekly subcutaneous CCR5 antagonist, leronlimab, is being investigated in combination with an OBR in HTE patients failing their current ARV regimen and as monotherapy maintenance in patients who are already virologically suppressed.^47^ An additional CD4 attachment inhibitor, UB-421, is under development and being evaluated for the treatment of patients with multidrug-resistant HIV and for an HIV cure. A new class of ARVS, maturation inhibitors, are also under development. GSK2838232 is a second-generation maturation inhibitor dosed once daily with a pharmacokinetic booster that is in phase IIa trials, with additional LA maturation inhibitors under development.

## Conclusion

Considerable progress has been made in the treatment of HTE PWH. However, as the incidence of drug resistance continues to increase, new targets are needed for salvage therapy. Appropriate identification of HTE PWH, management of resistance, and treatment using novel ARVs by clinicians treating PWH are critical to increase rates of virologic suppression and reduce the likelihood of development and spread of DRMs.

## References

[1] Cameron DW Heath-Chiozzi M Danner S , et al. Randomised placebo-controlled trial of ritonavir in advanced HIV-1 disease. The Advanced HIV Disease Ritonavir Study Group. Lancet 1998; 351: 543–549. 9492772 10.1016/s0140-6736(97)04161-5

[2] Hammer SM Squires KE Hughes MD , et al. A controlled trial of two nucleoside analogues plus indinavir in persons with human immunodeficiency virus infection and CD4 cell counts of 200 per cubic millimeter or less. AIDS Clinical Trials Group 320 Study Team. N Engl J Med 1997; 337: 725–733. 9287227 10.1056/NEJM199709113371101

[3] Spivack S Pagkalinawan S Samuel R , et al. HIV: how to manage heavily treatment-experienced patients. Drugs Context 2022; 11: 2021–9–1. doi: 10.7573/dic.2021-9-1. 10.7573/dic.2021-9-1 PMC8903874 35310298

[4] Bajema KL Nance RM Delaney JAC , et al. Substantial decline in heavily treated therapy-experienced persons with HIV with limited antiretroviral treatment options. AIDS 2020; 34: 2051–2059. 33055569 10.1097/QAD.0000000000002679 PMC7606534

[5] Pelchen-Matthews A Borges AH Reekie J , et al. Prevalence and Outcomes for Heavily Treatment-Experienced Individuals Living With Human Immunodeficiency Virus in a European Cohort. JAIDS 2021; 87: 806–817. 33587506 10.1097/QAI.0000000000002635

[6] HIV drug resistance: brief report 2024. Geneva: World Health Organization; 2024. https://iris.who.int/bitstream/handle/10665/376039/9789240086319-eng.pdf?sequence=1. Accessed March 1, 2024.

[7] Loosli T Hossmann S Ingle SM , et al. HIV-1 drug resistance in people on dolutegravir-based antiretroviral therapy: a cohort analysis. Lancet HIV 2023; 10: e733–e741. 37832567 10.1016/S2352-3018(23)00228-X PMC10913014

[8] Steegan K Macleaod WB Hans L , et al. Close monitoring of dolutegravir resistance in patients with laboratory confirmed dolutegravir exposure: observations from the 2022 national HIV drug resistance survey in South Africa. https://www.hivresistance.co.za/wp-content/uploads/2023/10/20230920-Steegan.pdf. Accessed March 24, 2024.

[9] Hsu RK Fusco JS Henegar CE , et al. Heavily treatment-experienced people living with HIV in the OPERA® cohort: population characteristics and clinical outcomes. BMC Infect Dis 2023; 23: 91. 36782125 10.1186/s12879-023-08038-w PMC9926692

[10] Bajema KL Nance RM Delaney JA , et al. Significant decline in heavily treatment experienced persons with HIV with limited antiretroviral treatment options in the US, 2000–2017. AIDS 2020; 34: 2051–2059. 33055569 10.1097/QAD.0000000000002679 PMC7606534

[11] Gupta S Granich R. HIV treatment in sub-Saharan Africa: delays in transition to dolutegravir. Poster session presented at: IAS Conference on HIV Science. The 11th Conference of the International AIDS Society. 18–21 July 2021, Berlin, Germany.

[12] The path that ends AIDS: UNAIDS Global AIDS Update 2023. Geneva: Joint United Nations Programme on HIV/AIDS; 2023. License: CC BY-NC-SA 3.0 IGO.

[13] Panel on Antiretroviral Guidelines for Adults and Adolescents. Guidelines for the Use of Antiretroviral Agents in Adults and Adolescents with HIV. Department of Health and Human Services. 2024. https://clinicalinfo.hiv.gov/en/guidelines/adult-and-adolescent-arv. Accessed January 1, 2024.

[14] Clutter DS Jordan MR Bertagnolio S , et al. HIV-1 drug resistance and resistance testing. Infect Genet Evol 2016; 46: 292–307. doi: 10.1016/j.meegid.2016.08.031. 27587334 10.1016/j.meegid.2016.08.031 PMC5136505

[15] Smith J Bansi-Matharu L Cambiano V , et al. Predicted effects of the introduction of long-acting injectable cabotegravir pre-exposure prophylaxis in sub-Saharan Africa: a modelling study. Lancet HIV 2023; 10: e254–e265. doi: 10.1016/S2352-3018(22)00365-4. 36642087 10.1016/S2352-3018(22)00365-4 PMC10065903

[16] Van Welzen BJ Van Lelyveld S Ter Beest G , et al. Virologic failure after switch to long-acting cabotegravir and rilpivirine injectable therapy: an in-depth analysis. Clin Infect Dis 2024; 79: 189–195. 38207125 10.1093/cid/ciae016 PMC11259215

[17] Margot NA Naik V VanderVeen L , et al. Resistance Analyses in Highly Treatment-Experienced People with Human Immunodeficiency Virus (HIV) Treated With the Novel Capsid HIV Inhibitor Lenacapavir. J Infect Dis 2022; 226: 1985–1991. doi: 10.1093/infdis/jiac364. 36082606 10.1093/infdis/jiac364

[18] Bansode V McCormack GP Crampin AC , et al. Characterizing the emergence and persistence of drug resistant mutations in HIV-1 subtype C infections using 454 ultra deep pyrosequencing. BMC Infect Dis 2013; 13: 52. 23363532 10.1186/1471-2334-13-52 PMC3740783

[19] McCluskey SM Seidner MJ Marconi VC. Management of Virologic Failure and HIV Drug Resistance. Infect Dis Clin North Am 2019; 33: 707–742. 31255384 10.1016/j.idc.2019.05.004 PMC6688946

[20] Madruga JV Cahn P Grinsztejn B , et al. Efficacy and safety of TMC125 (etravirine) in treatment-experienced HIV-1-infected patients in DUET1: 24-week results from a randomised, double-blind, placebo-controlled trial. Lancet 2007; 370: 29–38. 17617270 10.1016/S0140-6736(07)61047-2

[21] Lazzarin A Campbell T Clotet B , et al. Efficacy and safety of TMC125 (etravirine) in treatment-experienced HIV-1-infected patients in DUET2: 24-week results from a randomised, double-blind, placebo-controlled trial. Lancet 2007; 370: 39–48. 17617271 10.1016/S0140-6736(07)61048-4

[22] Steigbigel RT Cooper DA Kumar PN , et al. Raltegravir with optimized background therapy for resistant HIV-1 infection. N Engl J Med 2008; 359: 339–354. 18650512 10.1056/NEJMoa0708975

[23] Yazdanpanah Y Fagard C Descamps D , et al; ANRS 139 TRIO Trial Group. High rate of virologic suppression with raltegravir plus etravirine and darunavir/ritonavir among treatment-experienced patients infected with multidrug-resistant HIV: results of the ANRS 139 TRIO trial. Clin Infect Dis 2009; 49: 1441–1449. 19814627 10.1086/630210

[24] Nozza S Galli L Bigoloni A , et al. Durability and safety of a novel salvage therapy in R5-tropic HIV-infected patients: maraviroc, raltegravir, etravirine. J Acquir Immune Defic Syndr 2011; 56: e113–e115. 21350358 10.1097/QAI.0b013e31820a9ae4

[25] Tashima KT Smeaton LM Fichtenbaum CJ , et al. HIV Salvage Therapy Does Not Require Nucleoside Reverse Transcriptase Inhibitors: A Randomized, Controlled Trial. Ann Intern Med 2015; 163: 908–917. 26595748 10.7326/M15-0949 PMC4681296

[26] Capetti AF Sterrantino G Cossu M , et al. Salvage therapy or simplification of salvage regimens with dolutegravir plus ritonavir-boosted darunavir dual therapy in highly cART-experienced subjects: an Italian cohort. Antivir Therapy 2017; 22: 257–262. 10.3851/IMP3095 27661787

[27] Paton NI Musaazi J Kityo C , et al. Efficacy and safety of dolutegravir or darunavir in combination with lamivudine plus either zidovudine or tenofovir for second-line treatment of HIV infection (NADIA): week 96 results from a prospective, multicentre, open-label, factorial, randomised, non-inferiority trial. Lancet HIV 2022; 6: e381–e393. 10.1016/S2352-3018(22)00092-3 35460601

[28] Sammet S Touzeau-Romer V Wolf E , et al. The DoDo experience: an alternative antiretroviral 2-drug regimen of doravirine and dolutegravir. Infection 2023; 51: 1823–1829. 37526898 10.1007/s15010-023-02075-y PMC10665222

[29] Ramgopal MN Castagna A Cazanave C , et al. Efficacy, safety, and tolerability of switching to long-acting cabotegravir plus rilpivirine versus continuing fixed-dose bictegravir, emtricitabine, and tenofovir alafenamide in virologically suppressed adults with HIV, 12-month results (SOLAR): a randomised, open-label, phase 3b, non-inferiority trial. Lancet HIV 2023; 10: e566–e577. 37567205 10.1016/S2352-3018(23)00136-4

[30] Portilla J Arazo P Crusells J , et al. Dual therapy with darunavir/r plus etravirine is safe and effective as switching therapy in antiretroviral experienced HIV-patients. The BITER Study. J Int AIDS Soc 2014; 17: 19803. 25397547 10.7448/IAS.17.4.19803 PMC4225347

[31] Capetti AF Sterrantino G Cossu MV , et al. Switch to Dolutegravir plus Rilpivirine Dual Therapy in cART-Experienced Subjects: An Observational Cohort. PLoS ONE 2016; 11: e0164753. 27741309 10.1371/journal.pone.0164753 PMC5065232

[32] Huhn GD Tebas P Gallant J , et al. A Randomized, Open-Label Trial to Evaluate Switching to Elvitegravir/Cobicistat/Emtricitabine/Tenofovir Alafenamide Plus Darunavir in Treatment-Experienced HIV-1-Infected Adults. J Acquir Immune Defic Syndr 2017; 74: 193–200. 27753684 10.1097/QAI.0000000000001193 PMC5228611

[33] Podzamczer D Imaz A Lopez-Lirola A , et al. Switching to bictegravir/emtricitabine/tenofovir alafenamide (BIC/FTC/TAF) plus darunavir/cobicistat in heavily antiretroviral-experienced, virologically suppressed HIV-infected adults receiving complex regimens. J Antimicrob Chemother 2023; 78: 2696–2701. 37725999 10.1093/jac/dkad285

[34] Coffman C Brizzi MB Adjei Z , et al. Simplifying Antiretroviral Treatment Regimens in Patients with Multi-Drug Resistant HIV. Poster session presented at: ACCP Global Conference. 15-18 October 2022, San Francisco, CA.

[35] University of Liverpool. HIV Drug Interactions. https://www.hiv-druginteractions.org/checker. Accessed September 15, 2024.

[36] Orkin C Cahn P Castagna A , et al. Opening the door on entry inhibitors in HIV: Redefining the use of entry inhibitors in heavily treatment experienced and treatment-limited individuals living with HIV. HIV Med 2022; 23: 936–946. 35293094 10.1111/hiv.13288 PMC9546304

[37] Vojnoy L Carmona S Zeh C , et al. The performance of using dried blood spot specimens for HIV-1 viral load testing: A systematic review and meta-analysis. PloS Med 2022; 19: e1004076. 35994520 10.1371/journal.pmed.1004076 PMC9447868

[38] Consolidated guidelines on HIV prevention, testing, treatment, service delivery and monitoring: recommendations for a public health approach. Geneva: World Health Organization; 2021. License: CC BY-NC-SA 3.0 IGO. 34370423

[39] TROGARZO (ibalizumab-uiyk) [package insert]. Montréal, Québec Canada: Theratechnologies Inc.; 2021.

[40] Emu B Fessel J Schrader S , et al. Phase 3 study of ibalizumab for multidrug-resistant HIV-1. N Engl J Med 2018; 379: 645–654. 30110589 10.1056/NEJMoa1711460

[41] RUKOBIA (fostemsavir) [package insert]. Research Triangle Park, NC, USA: ViiV Healthcare; 2020.

[42] Aberg JA Shepherd B Wang M , et al. Week 240 Efficacy and Safety of Fostemsavir Plus Optimized Background Therapy in Heavily Treatment-Experienced Adults with HIV-1. Infect Dis Ther 2023; 12: 2321–2335. 37751019 10.1007/s40121-023-00870-6 PMC10581994

[43] SUNLENCA (lenacapavir) [package insert]. Foster City, CA, USA: Gilead Sciences Inc.; 2022.

[44] Segal-Maurer S DeJesus E Stellbrink HJ , et al. Capsid Inhibition with Lenacapavir in Multidrug-Resistant HIV-1 Infection. N Engl J Med 2022; 386: 1793–1803. 35544387 10.1056/NEJMoa2115542

[45] Study to Compare Bictegravir/Lenacapavir Versus Current Therapy in People With HIV-1 Who Are Successfully Treated With a Complicated Regimen (ARTISTRY-1). NCT05502341. ClinicalTrials.gov, 2022. Accessed on March 23, 2024. https://classic.clinicaltrials.gov/ct2/show/NCT05502341.

[46] Rana AI Bao Y Zheng L , et al. Long-Acting Injectable CAB/RPV is Superior to Oral ART in PWH With Adherence Challenges: ACTG A5359. Poster session presented at: Conference on Retroviruses and Opportunistic Infections. 3–6 March 2024, Denver, CO, USA.

[47] Temereanca A Ruta S. Strategies to overcome HIV drug resistance-current and future perspectives. Front Microbiol 2023; 14: 1133407. 36876064 10.3389/fmicb.2023.1133407 PMC9978142

